# Practical Treatise on Fractures and Dislocations

**Published:** 1872-01

**Authors:** 


### Books Reviewed.

*Practical Treatise on Fractures and Dislocations.* By Frank Hastings Hamilton, A M., M.D., L.L.D. Henry C. Lea, Philadelphia, 1871.

In this, the fourth edition, the author says: Discussions which occupied considerable space in previous editions have been omitted in the present; such for example, as that relating to the value of certain specimens claiming to represent bony union after intra-capsular fractures of the neck of the femur. Many obsolete forms of apparatus have also been excluded. Nearly one-fourth of the whole number of illustrations have been changed, in most cases by the substitution of original wood cuts. This he says has been done to make room for more practical observations. In the preface the author gives a well deserved and truthful compliment to Dr. Bigelow of Boston, and speaks of having introduced into his work " several of the beautiful illustrations contained in Dr. Bigelow's treatise on the *Mechanism of Dislocation and Fracture of the Hip*." He says: " Since Sir Astley Cooper wrote, probably no man has thrown so much light upon the subject of hip-joint accidents, or contributed so much toward an accurate and systematic plan of treatment as the distinguished Boston surgeon."

This work by Dr. Hamilton is so thoroughly before the profession that after hinting the changes which have been made little further need be said.

The present volume comes to us greatly enlarged and improved, and highly as we prized the work in its former editions we regard the present as so much improved in all respects, as to have nearly lost its identity and assumed the features of a new and complete treatise on fractures and dislocations. Perhaps there is no one standard surgical work consulted more frequently and with deeper interest than this, and it is not saying too much of it to affirm that it has been of incomparable value to surgeons in obtaining satisfactory results, as well as in determining what is really to be expected after fracture and dislocation.

The work in its first edition was written in this city, and many of the original observations upon which his conclusions rest, were made in Buffalo and vicinity. It is natural that we claim somewhat on account of the birth place of the work; it has grown vigorously and healthy, and is now offered to the profession in matter and style of which not only the author, but the American profession may well be proud.

As already intimated, to this work surgeons and judges and juries all turn, finding it always faithful to fact, always sustained by careful clinical observation, always on the side of the skillful surgeon, always in the right; a work no surgeon is safe without owning and reading.

